# IRS1 deficiency protects β-cells against ER stress-induced apoptosis by modulating sXBP-1 stability and protein translation

**DOI:** 10.1038/srep28177

**Published:** 2016-07-05

**Authors:** Tomozumi Takatani, Jun Shirakawa, Michael W. Roe, Colin A. Leech, Bernhard F. Maier, Raghavendra G. Mirmira, Rohit N. Kulkarni

**Affiliations:** 1 Islet Cell and Regenerative Biology, Joslin Diabetes Center, Department of Medicine, Brigham and Women’s Hospital, Harvard Stem Cell Institute, Harvard Medical School, Boston, MA, USA.; 2 Department of Medicine, State University of New York (SUNY), Upstate Medical University, Syracuse, NY, USA; 3 Department of Pediatrics and Herman B Wells Center for Pediatric Research, Indiana University School of Medicine, Indianapolis, IN, USA; 4 Department of Cellular and Integrative Physiology, Department of Biochemistry and Molecular Biology, Department of Medicine, Indiana University School of Medicine, Indianapolis, IN, USA.

## Abstract

Endoplasmic reticulum (ER) stress is among several pathological features that underlie β-cell failure in the development of type 1 and type 2 diabetes. Adaptor proteins in the insulin/insulin-like-growth factor-1 signaling pathways, such as insulin receptor substrate-1 (IRS1) and IRS2, differentially impact β-cell survival but the underlying mechanisms remain unclear. Here we report that β-cells deficient in IRS1 (IRS1KO) are resistant, while IRS2 deficiency (IRS2KO) makes them susceptible to ER stress-mediated apoptosis. IRS1KOs exhibited low nuclear accumulation of spliced XBP-1 due to its poor stability, in contrast to elevated accumulation in IRS2KO. The reduced nuclear accumulation in IRS1KO was due to protein instability of Xbp1 secondary to proteasomal degradation. IRS1KO also demonstrated an attenuation in their general translation status in response to ER stress revealed by polyribosomal profiling. Phosphorylation of eEF2 was dramatically increased in IRS1KO enabling the β-cells to adapt to ER stress by blocking translation. Furthermore, significantly high ER calcium (Ca^2+^) was detected in IRS1KO β-cells even upon induction of ER stress. These observations suggest that IRS1 could be a therapeutic target for β-cell protection against ER stress-mediated cell death by modulating XBP-1 stability, protein synthesis, and Ca^2+^ storage in the ER.

Understanding the mechanism(s) underlying β-cell dysfunction is important to design therapeutic approaches for both type 1 and type 2 diabetes. Over the last decade, considerable evidence has accumulated pointing to critical roles for growth factor signaling proteins, such as insulin receptor substrate (IRS1) and IRS2, in the regulation of islet cell growth and function^1^,^2^,^3^. While genetic approaches have revealed that IRS1 and IRS2 signaling pathways differentially impact β-cell growth, survival, and/or function^4^,^5^,^6^,^7^,^8^,^9^, the distinct roles of these two proteins in pathophysiological conditions have not been fully explored. Endoplasmic reticulum (ER) stress, caused by dysregulation of ER homeostasis, contributes to β-cell apoptosis in the development of type 2 diabetes^10^,^11^. In stressed cells the activation of the unfolded protein response (UPR) regulates their adaptation to ER stress. When the UPR fails to maintain ER homeostasis, in the face of unfolded protein overload, apoptosis ensues. The UPR involves the activation of three pathways including IRE1α, PERK and ATF6. In response to ER stress, IRE1α activates XBP-1 through unconventional splicing of XBP-1 mRNA, followed by translocation of spliced XBP-1 (sXBP1) into the nucleus for the induction of chaperone proteins which restore ER homeostasis^12^. PERK suppresses general protein synthesis through phosphorylation of eIF2α in response to ER stress while the translation of selected UPR mRNAs such as ATF4 is enhanced under ER stress^13^. It is notable that proteins in the growth factor or nutrient signaling pathway crosstalk with other ER stress signaling pathways in β-cells and other tissues^1^,^2^,^3^,^13^,^14^,^15^. For example, p85, a regulatory subunit of PI3K that mediate insulin/IGF-1 signaling, regulates ER stress in the hepatocyte by modulating XBP-1 nuclear translocation^13^,^14^. Moreover, IGF-1 signaling, whose downstream components are shared with insulin signaling, augments the adaptive capacity of the ER via increased compensatory mechanisms such as IRE1α, PERK and ATF6-mediated arms of the ER stress signaling pathway in fibroblasts^15^. Since inhibitors of MEK, PI3K, JNK, p38, protein kinase A, protein kinase C and STAT3 do not inhibit the effects of IGF1 on ER stress, it is likely that as yet unidentified proteins are operational in IR/IGF1R signaling in the context of ER stress^15^. Together these data point to a role for growth factor signaling in the regulation of ER stress in β-cells.

Mice with a deficiency of IRS1 exhibit hyperplastic islets due to insulin resistance while IRS2KO mice exhibit islet hypoplasia^4^,^5^. Previous studies have revealed the intrinsic roles played by the substrates in β-cells in contributing to the phenotypic differences between IRS1KO and IRS2KO mice^16^,^17^. However, the significance of IRS1 or IRS2 specifically under conditions of ER stress in β-cells has not been fully investigated. We therefore evaluated ER stress in cell lines lacking either IRS1 or IRS2^4^,^18^. Here we report that IRS1KO β-cells are resistant to ER stress-mediated cell death by modulating the IRE1α-XBP-1 arm of the unfolded protein response, protein translation and Ca^2+^ flux in ER. In contrast, exposure of IRS2 KO β-cells to ER stress leads to increased accumulation of XBP-1 in the nucleus while maintaining similar translation status and Ca^2+^ flux as control β-cells. These findings shed light on potential mechanism(s) underlying the phenotypic differences between β-cells lacking IRS1 or IRS2.

## Results

### Lack of IRS1 prevents β-cell apoptosis induced by ER stress

**Figure 1:**
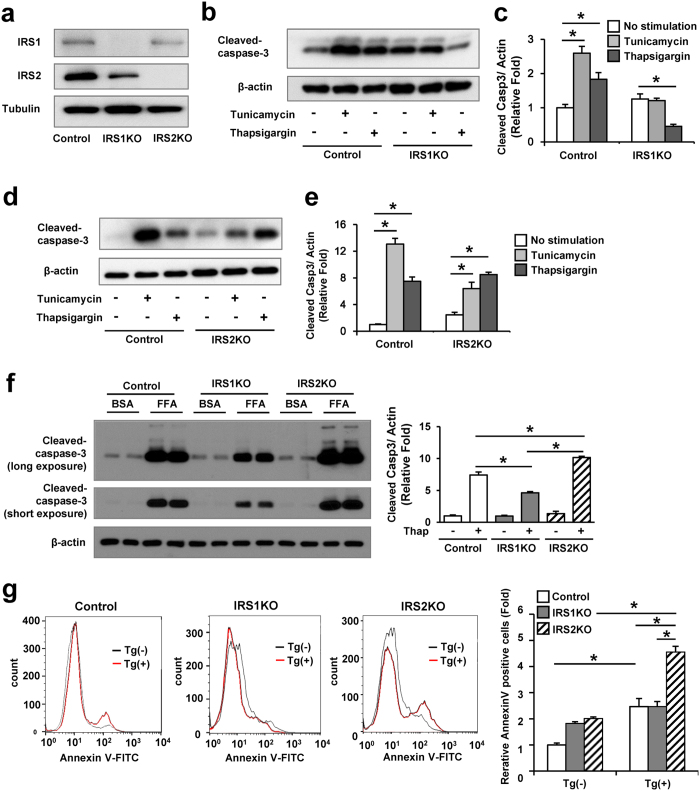
IRS1KO β-cells exhibit resistance to ER stress-mediated cell death. (**a**) Immunoblot confirms absence of IRS1 and IRS2 compared to control β-cell lines. (**b–e**) Immunoblot of cleaved caspase-3 in control, IRS1KO, and IRS2KO β-cells incubated with tunicamycin (2 μg/ml) or thapsigargin (100 nM) for 8h Data are means ± SEM, n = 3. *P < 0.05. (**f**) Immunoblot of cleaved caspase-3 in control, IRS1KO, and IRS2KO β-cells incubated with 0.5 mM palmitate for 24 h. Data are means ± SEM, n = 3. *P < 0.05. (**g**) Results of FACS analysis for Annexin V-positive cells in control, IRS1KO, or IRS2KO β-cells incubated with thapsigargin (Tg) (100 nM) for 24 h. Data are means ± SEM, n = 3. *P < 0.05.

To examine the relevance of IRS1 and IRS2 in ER stress-induced apoptosis, we stimulated IRS1KO or IRS2KO β-cells^4^,^18^ (Fig. 1a), with two widely used stimuli namely, tunicamycin or thapsigargin for 8 hours. The level of cleaved caspase-3 after tunicamycin or thapsigargin stimulation was increased in control and IRS2KO β-cells, but not in IRS1KO β-cells (Fig. 1b–e). A similar decrease in cleaved caspase-3 levels in IRS1KO β-cells was also evident in response to free fatty acid (palmitate)-induced ER stress (Fig. 1f). In contrast, IRS2KO β-cells demonstrated increased cleaved caspase-3 levels upon induction of free fatty acid-induced ER stress (Fig. 1f). We also measured apoptosis by flow cytometry using Annexin V-FITC. Both control and IRS2KO showed an increased number of apoptotic cells in response to ER stress induced by thapsigargin or tunicamycin in contrast to a lack of significant increase in IRS1KO β-cells (Fig. 1g and see Supplementary Fig. S1). These results suggest that IRS1KO β-cells are resistant to ER stress-induced apoptosis compared with control β-cells or IRS2KO β-cells.

### Reduced nuclear accumulation of XBP-1 in IRS1KO β-cells

**Figure 2:**
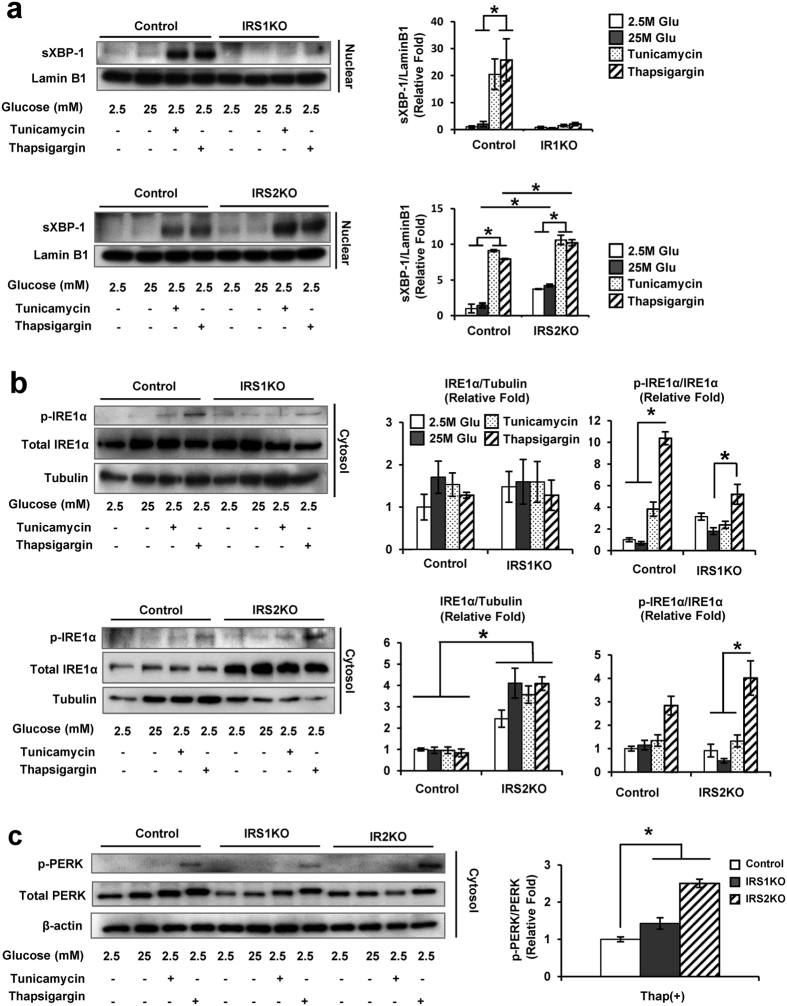
IRS1KO β-cells shows decreased nuclear accumulations of sXBP-1. (**a–c**) Control, IRS1KO, and IRS2KO β-cells incubated with low glucose (2.5 mM), high glucose (25 mM), tunicamycin (2 μg/ml), or thapsigargin (100 nM) for 4 h after overnight starvation. (**a**) Immunoblot of sXBP-1 and lamin B1 (loading control) in nuclear fractions. Data are means ± SEM, n = 3. *P < 0.05. (**b**) Immunoblot of p-IREα, IRE1α and tubulin (loading control) using cytosolic fractions. Data are means ± SEM, n = 3. *P < 0.05. (**c**) Immunoblot of p-PERK, PERK, and β-actin using cytosolic fractions. Data are means ± SEM, n = 3. *P < 0.05.

**Figure 3:**
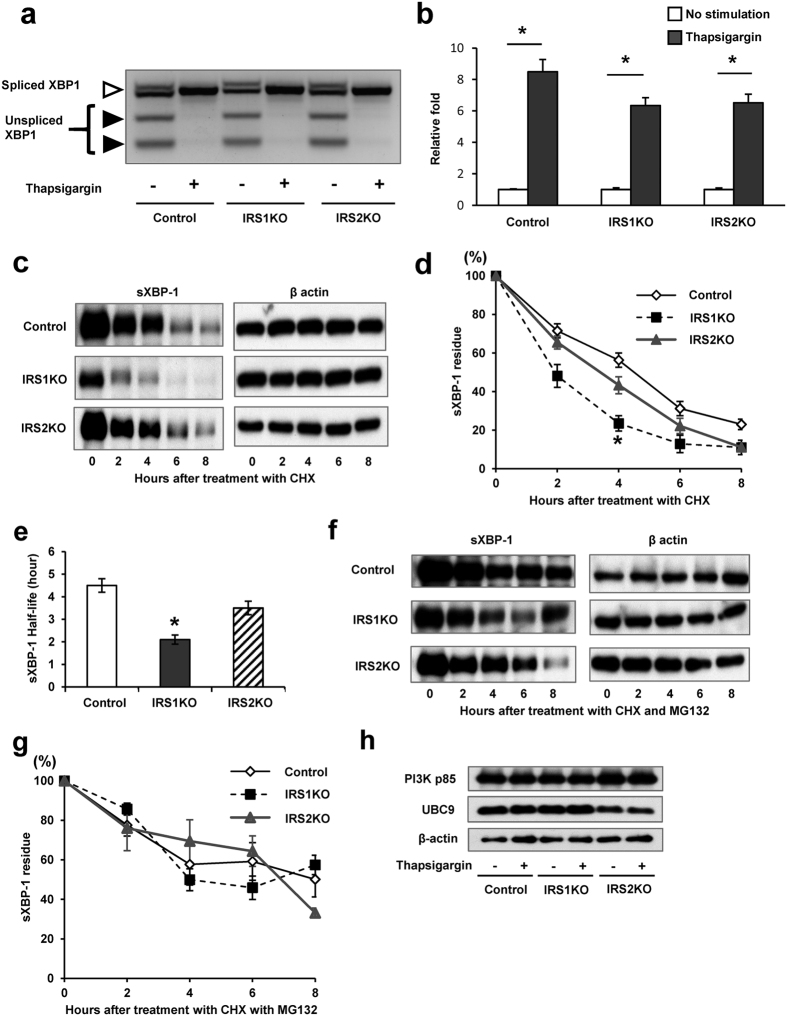
Decreased stability of sXBP-1 in IRS1KO β-cells. (**a**) XBP-1 splicing assay: 2% Agarose gel electrophoresis of PstI digested PCR fragments from control, IRS1KO or IRS2KO β-cells stimulated with thapsigargin for 2 h. (**b**) Relative expression of the active form of XBP-1 mRNA in control, IRS1KO or IRS2KO β-cells stimulated with thapsigargin (100 nM) for 2 h. (**c,d**) CHX assay using control, IRS1KO, or IRS2KO β-cells. Cells transfected and expressing sXBP-EGFP fusion protein were treated with CHX (100 μg/ml) and harvested at indicated times. Data are means ± SEM, n = 3. *P < 0.05 vs control β-cells. (**e**) Half-life of sXBP1 was measured using data from the CHX assay. Data are means ± SEM, n = 3. *P < 0.05 vs control β-cells. (**f,g**) CHX assay performed with MG132 (5 μM). Data are means ± SEM, n = 3. (**h**) Immunoblot of PI3K p85, UBC9, and β-actin in control, IRS1KO, and IRS2KO β-cells incubated with or without thapsigargin for 4 h.

Next, we evaluated the expression of ER stress markers in the β-cells following treatment with tunicamycin or thapsigargin for 4 hours to check the acute response. We used high (25 mM) glucose as an additional stimulus since it has been reported to enhance ER stress in β-cells^19^,^20^. Evaluation of the IRE1α-XBP-1 branch of UPR revealed that the accumulation of sXBP-1 protein in the nuclear fraction under ER stress was reduced in IRS1KO β-cells, but was increased in IRS2KO β-cells, compared with controls (Fig. 2a). To investigate the reduction in sXBP-1 in IRS1KO β-cells, we examined IRE1α phosphorylation. Treatment with tunicamycin or thapsigargin phosphorylated IRE1α to a similar degree in all three groups (Fig. 2b). Phospholyration of PERK was not significantly altered in IRS1KO β-cells, but increased in IRS2KO β-cells compared to controls (Fig. 2c). Evaluation of XBP1 mRNA splicing in β-cells, using a PCR-based^21^ assay, revealed normal splicing in all groups including IRS1KO β-cells (Fig. 3a). Further, no significant changes were evident in the ratio of spliced form of XBP1 measured by qPCR^22^ (Fig. 3b), suggesting that the intact splicing of XBP1 is unlikely to contribute to the reduced nuclear accumulation of sXBP1 in IRS1KO β-cells.

### IRS1 deficiency in β-cell promotes sXBP-1 instability

Previous reports indicate that the stability of sXBP1 is important for the UPR and is regulated by several factors including PI3K^13^,^14^. To explore whether protein degradation is involved in the apparent reduction in nuclear accumulation of sXBP1 observed in IRS1KO β-cells, we performed cycloheximide (CHX) chase assay to measure the half-life of sXBP1 in each group. IRS1KO β-cells exhibited a significantly lower residue of sXBP-1 4 hours after CHX treatment while IRS2KO β-cells showed a degradation pattern that was similar to control β-cells (Fig. 3c,d). Notably, the stability of β-actin, an internal control protein, showed no significant differences between the three groups up to 8 hours (Fig. 3c and see Supplementary Fig. S2a). Control β-cells and IRS2KO β-cells demonstrated comparable levels of stability of sXBP-1 protein, with half-lives ranging from 3.5 to 4.5 hours while it was significantly shorter (~2 hours) in IRS1KO β-cells (Fig. 3e). To explore this further and considering sXBP-1 is known to be degraded through a proteasome pathway^21^, we examined the half-life of sXBP-1 following treatment with CHX in the presence of a proteasome inhibitor, MG132. The shortened half-life of sXBP-1, but not β-actin, was blunted following treatment of IRS1KOs with MG132 over the 8 h duration (Fig. 3f,g and Supplementary Fig. S2b), suggesting that sXBP-1 exhibits greater proteasomal degradation in these cells compared to control or IRS2KO β-cells. Examination of expression of binding proteins which stabilize sXBP-1, such as PI3K and UBC9^13^,^14^,^23^, did not show significant differences between groups (Fig. 3h), implying the mechanism is independent of these pathways. Analyses of uqibuitination of XBP1, following co-transfection of XBP1 and ubiquitin in control or IRS1KO β-cells, revealed no differences between groups (See supplementary Fig. S3a). We next reasoned that the altered stability of sXBP1 in the resistant IRS1KO β-cells is potentially secondary to changes in phosphorylation of sXBP1. We therefore examined two sites on sXBP1 phosphorylated by p38 MAPK, serine 61 or threonine 48^24^. While control β-cells showed a significant phosphorylation of p(ser61)-sXBP1 in response to stimulation with thapsigargin (See Supplementary Fig. S3b), the basal levels of p(ser61)-sXBP1 was already elevated in IRS1KOs and stimulation with thapsigargin did not increase it further. Furthermore, no significant differences were observed in either group in p(thr48)-sXBP1 (See Supplementary Fig. S3b). These data suggest that β-cells lacking IRS1 are resistant and exhibit poor phosphorylation of sXBP1 under ER stress states; and this does not necessitate nuclear accumulation of sXBP-1.

### Attenuated translation in response to ER stress in IRS1KO β-cells due to increased phosphorylation of eEF2

**Figure 4:**
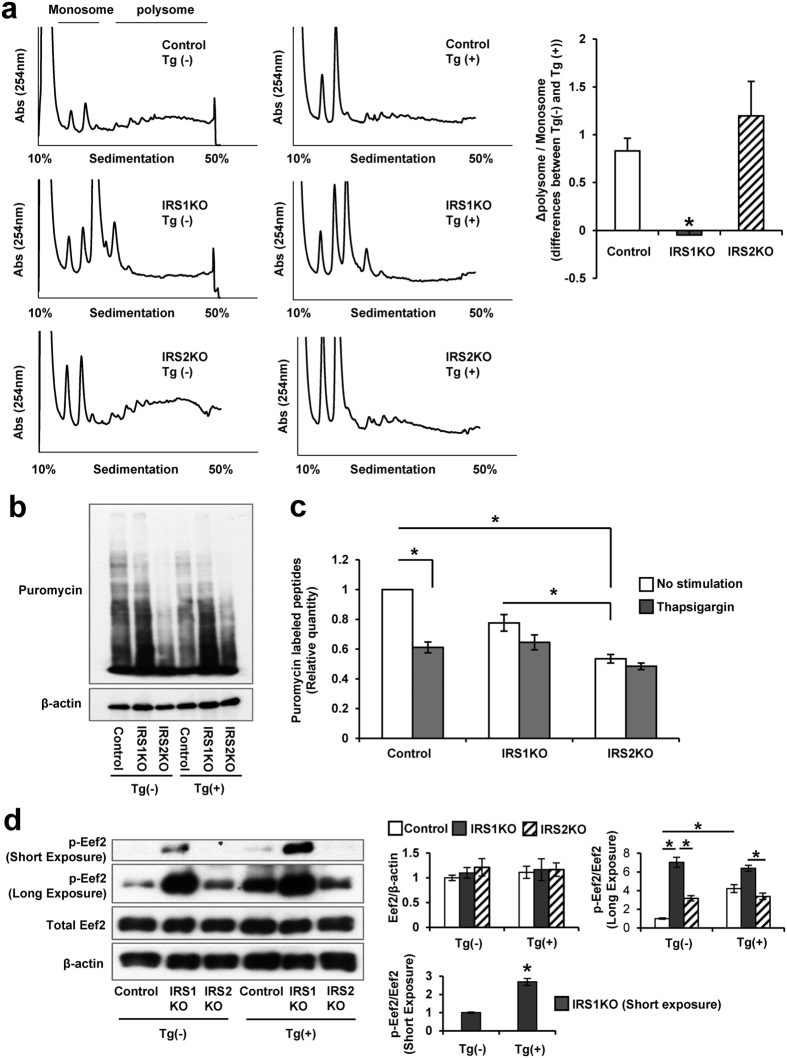
Attenuated translation in IRS1KO β-cells in response to ER stress. (**a**) Polysome profiles for control, IRS1KO or IRS2KO β-cells incubated with vehicle or thapsigargin (Tg) (10 nM) for 4 h. Representative polysome profiles are shown (left panel). The differences in the P/M ratio in cells ± thapsigargin (10 nM) is shown in the right panel. The data relate to ratio of polysomal to monosomal (40S, 60S, and 80S) fractions. Data are means ± SEM, n = 4. *P < 0.05. (**b,c**) Immunoblot of control, IRS1KO and IRS2KO β-cells after teatment with puromycin. Data are means ± SEM, n = 3. *P  < 0.05. (**d**) Immunoblot of p-eEF2, eEF2 and β-actin in control β-cells, and IRS2KO β-cells incubated with vehicle or thapsigargin (100 nM) for 8 h. Data are means ± SEM, n = 3. *P < 0.05.

Insulin and ER stress both regulate global translation with the latter acting via the PERK-eIF2α pathway^23^,^25^. We therefore investigated whether translational control is modulated in IRS deficient cells by polyribosomal profiling (PRP). As expected, thapsigargin treatment caused a dramatic reduction in the ratio of polysome to monosome (P/M) in both control and IRS2KO β-cells (Fig. 4a, left panels). However, IRS1KO β-cells showed minimal changes in translation, as indicated by the significantly small change in the P/M ratio (calculated as difference between thapsigargin treated and untreated conditions), compared with the change observed in control or IRS2KO β-cells (Fig. 4a, right panel). Both IRS1KO and IRS2KO β-cells tended to have a lower puromycin incorporation in the basal state and the incorporation did not alter significantly even after stimulation with thapsigargin in contrast to the effect in control β-cells (Fig. 4b,c). Thus, the change in translational state induced by the stressed condition was lower in IRS1KO β-cells. A further distinguishing feature between the IRS1KO and IRS2KO β-cells was the significantly elevated phosphorylation of eukaryotic elongation factor 2 (eEF2) in the former in the presence or absence of thapsigargin stimulation (Fig. 4d). The phosphorylation of mTOR, which is thought to reduce the phosphorylation of eEF2^26^, was also attenuated in IRS1KO β-cells than control β-cells (See Supplementary Fig. S4). However, phosphorylation of 4EBP1 was not significantly different between groups (See Supplementary Fig. S4). The increased phosphorylated eEF2 in IRS1KO β-cells indicates a blockade of translation elongation under ER stress in addition to inhibition of translation initiation. In this context it is notable that eEF2K, a kinase that phosphorylates eEF2, also regulates autophagy^27^,^28^,^29^. Therefore, to examine the effects of eEF2K activity on autophagy we assessed expression of p62 and LC3. Remarkably, p62 was significantly decreased in IRS1KO β-cells compared with control or IRS2KO β-cells (See Supplementary Fig. S5a); this was associated with a decreased level of LC3II and autophagic vacuoles in IRS1KO β-cells (See Supplementary Fig. S5b,c). These data implicate an increased flux of proteins in the autophagy process which is consistent with alterations in eEF2K activity in IRS1KO β-cells that might contribute to the resistance in ER-stress mediated cell death.

### Ca^2+^ storage in ER is preserved under ER stress conditions in IRS1KO β-cells

**Figure 5:**
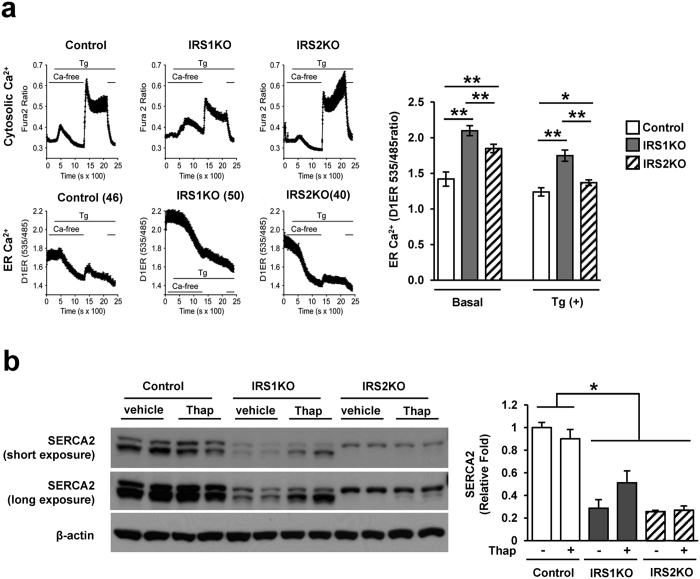
Ca^2+^ storage of ER in IRS1KOs is higher than control or IRS2KO β-cells. Ca^2+^ in cytosol was measured using Fura-2 and Ca^2+^ in ER was measured using FRET-based probe D1ER cameleon in the basal state and after thapsigargin stimulation (100 nM for 1000 sec). Representative Ca^2+^ measurements in cytosol and ER (left panel). Quantitative ER Ca^2+^ levels of the average value prior to thapsigargin stimulation (Basal) and the minimum value after addition of thapsigargin (Tg(+)) (right panel). Data are means ± SEM, n = 43 for control, n = 50 for IRS1KO, and n = 40 for IRS2KO β-cells. *P < 0.05, **P < 0.01.

We next investigated Ca^2+^ storage since its depletion in the ER acts as a trigger for apoptosis induced by ER stress^30^,^31^. Cytosolic and ER Ca^2+^ concentration were evaluated in the presence or absence of ER stress in control, IRS1KO, or IRS2KO β-cells. Cytosolic Ca^2+^ was low in IRS1KO β-cells compared to control or IRS2KO β-cells (Fig. 5a). Following thapsigargin stimulation Ca^2+^ depletion from ER was observed in all β-cells (Fig. 5a). ER Ca^2+^ was significantly higher in both basal and thapsigargin stimulated conditions in IRS1KO compared to control or IRS2 KO β-cells (Fig. 5a). The expression of sarco/endoplasmic reticulum Ca^2+^ ATPase 2 (SERCA2) was decreased in both IRS1KO and IRS2KO compared to control β-cells (Fig. 5b). The reduced cytosolic Ca^2+^ influx and reduced SERCA2 expression in IRS1KO β-cells are consistent with our previous study^17^. These changes in cytosolic and ER Ca^2+^ concentration in the IRS-1KOs appear independent of the morphology of their mitochondria which did not differ from control β-cells (See Supplementary Fig. S6). The increased Ca^2+^ storage in ER under ER stress in IRS1KO β-cells might be related to the attenuated apoptosis.

## Discussion

The unfolded protein response (UPR) is a cellular reaction to fluctuations in the normal functioning of the ER^32^. The pancreatic β-cells have a well-developed ER that is necessary to accomplish their most significant biological function of consistently and efficiently secreting insulin and other glycoproteins for appropriate maintenance of cell and organismal homeostasis. Considering the large volume of protein synthesis the β-cells are highly sensitive to alterations in ER function and consequently severe and/or long-term ER stress has been implicated as a trigger leading to β-cell dysfunction and death^11^.

Among proteins in the insulin signaling pathway PI3K is important to mitigate high fat diet induced ER stress in the liver by stabilizing XBP1^13^,^14^. Defects in PI3K lead to a reduced nuclear accumulation of XBP1 and consequent defects in glucose homeostasis^13^,^14^. Our data indicate that deficiency of IRS1 destabilizes XBP-1 and in turn protects β-cells from ER stress mediated apoptosis. These data are consistent with a previous report showing that lack of PI3K protects β-cells from ER stress mediated apoptosis in the Akita mouse that harbors a C96Y mutation in the insulin-2 gene^33^. Interestingly, lack of IRS2 did not affect sXBP-1 stability, suggesting that IRS1 and IRS2 have distinct roles in modulating the IRE1α-XBP-1 arm of the UPR.

Although the IRS1KO β-cells were resistant to ER-stress induced apoptosis, intriguingly, we observed a trend towards increased cleaved caspase-3 levels and Annexin V-positive cells in the basal state in IRS1KO β-cells. In contrast this elevation was not evident when the β-cells were cultured in the presence of BSA in the free fatty acid experiments (Fig. 1f). Whether the different culture conditions regulated the dynamics of proliferation versus apoptosis in the cells lacking IRS1 requires further investigation.

Annexin V-positive cells were significantly increased, whereas cleaved caspase-3 levels were not altered, under ER stress induced by tunicamycin or thapsigargin in IRS2KO β-cells compared to control β-cells. In contrast, palmitate-induced ER stress led to a significant increase in both Annexin V-positive cells and cleaved caspase-3 levels in IRS2KO. This apparent discrepancy between the expression of cleaved caspase-3 levels and Annexin V-positivity in response to different stimuli promoting ER stress might be secondary to activation of caspase-independent apoptotic pathways by IRS2 and/or the time course of treatment used in this study. Since thapsigargin is a non-competitive inhibitor of SERCA, a decrease in expression of the sarco/endoplasmic reticulum Ca^2+^-ATPase in IRS2KO β-cells might underlie the differences in susceptibility to ER stress. It is also possible that the increased total IRE1α in the basal state in IRS2KOs promotes nuclear accumulation of XBP-1 and increases susceptibility to ER stress-mediated cell death.

The lack of differences, between control and IRS1KO β-cells, in the expression of PI3K or UCB9 which are known to stabilize sXBP-1, suggests participation by other factors; for example, a non-canonical pathway that has been implicated in the anti-apoptotic activity of IGF1 during ER stress^15^ downstream of IRS1 could account for sXBP-1 instability in IRS1KO β-cells. In addition to lack of stability of sXBP-1, we observed altered protein translation in IRS1KO β-cells. Considering protein burden in secreting cells is known to promote ER stress, suppression of protein synthesis is an important adaptation to the stress response^34^. Although PERK-eIF2α is a major regulator of protein synthesis during ER stress the lack of differences in the increase in PERK phosphorylation after ER stress between control and IRS1KO β-cells indicates that this pathway is unlikely to contribute to the differences in protein translation between the cell types. On the other hand, translation status as measured by PRP, was less affected in IRS1KO β-cells in response to ER stress, suggesting that an elongation block through eEF2 inactivation is one potential contributor to the difference in apoptosis between groups. ER stress also induces 4EBP1 which reduces protein synthesis through an initiation block and alleviates ER stress burden to promote β-cell survival^35^. However, IRS1KO β-cells did not exhibit a significant increase in 4EBP1 (See Supplementary Fig. S4) implying this pathway is unlikely to be responsible for alterations in protein translation in IRS1KO β-cells.

Although ER stress has been generally linked to translation initiation, translation elongation also plays a role in response to diverse stressors to regulate ER stress-induced cell death^36^,^37^,^38^,^39^,^40^. eEF2, a member of the GTP-binding translation elongation factor family, is an essential protein involved in the movement of ribosomes along mRNAs during translation elongation and is inactivated by its phosphorylation^41^. Since IRS1KO β-cells harbor highly phosphorylated eEF2 both in the basal state and upon induction of ER stress, the blockade of translation elongation may contribute to increased survival of β-cells when IRS-1 is lacking. Increased phosphorylation levels of eEF2 in cardiomyocytes is correlated with the protective effect of AMPK on cell survival under hypoxic stress^42^, providing an example for the significance of eEF2 phosphorylation in anti-apoptotic pathways. One possibility for the increased phosphorylation of eEF2 in control and IRS1KO β-cells is that eukaryotic elongation factor 2 kinase (eEF2K), a protein kinase that phosphorylates eEF2, is normally suppressed by IRS1. Because mTOR-mediated inhibition of eEF2K induces dephophorylation and activation of eEF2, the reduced phosphorylation of mTOR in IRS1KO β-cells might also contribute to the phosphorylation of eEF2 in this study. It is worth noting that mTOR and eEF2K have been implicated in regulation of autophagy^43^,^44^, so that eEF2K could function to promote anti-apoptosis in IRS1KO β-cells. The significant difference in autophagic status between control and IRS1KO β-cells in this study, along with our previous observation of altered autophagic vacuoles in the endocrine pancreas of IRS1KO animals, supports this possibility^4^.

Several studies report that decreased Ca^2+^ concentration in ER triggers apoptosis^30^,^31^. In β-cells, depletion of ER Ca^2+^ is critical for cell death under ER stress through the activation of calpain-2^45^. IRS1 also directly interacts with sarco/endoplasmic reticulum Ca^2+^-ATPase (SERCA)-3^46^ and we have previously reported that islets lacking IRS-1 exhibit altered insulin secretory patterns due decreased SERCA 2b and 3 expression^4^,^17^. In fact, SERCA2 expression is involved in ER Ca^2+^ levels and survival under ER stress in β cells^47^,^48^. The reduced expression of SERCA2 might contribute to an increase in ER Ca^2+^ concentration in IRS1KOs. Since IRS1KO demonstrates a significantly smaller increase in intracellular Ca^2+^ concentration which in turn is accompanied by reduced insulin secretion in response to glucose and arginine^17^, the interplay between ER Ca^2+^ and cytosolic Ca^2+^ spikes and its impact on secretion versus cell death during ER stress is worth pursuing.

The observations in this study are consistent with an *in vivo* study where we reported that IRS1KO islets grafted into IRS1KO mice showed reduced apoptosis^16^. Although mice with global deletion of IRS1 manifest large islets^4^, the effects of ER stress *in vivo* and the influence of factors secondary to peripheral insulin resistance requires careful investigation. For example, it will be interesting to examine how absence of IRS1 or IRS2 in ER-stress induced beta cells *in vivo* is impacted by SerpinB1, a protease inhibitor secreted by the insulin resistant liver^49^, and potentially proteins in other pathways such as osteoprotegerin^50^ and Dyrk1a^51^.

**Figure 6:**
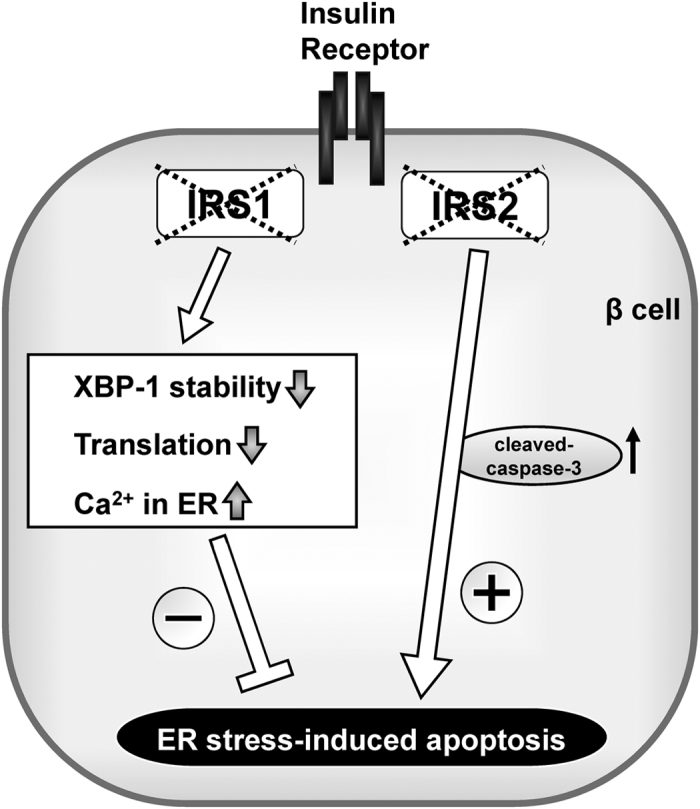
Schematic showing a role for IRS1 in the regulation of translation, sXBP-1 stability and Ca^2+^ storage in ER under ER stress conditions in β-cells. The deficiency of IRS1 leads to decreased translation, instability of sXBP-1 and preserved Ca^2+^ storage in ER, which leads to resistance to ER stress-induced β-cell apoptosis. In contrast, IRS2 deficient β-cells are vulnerable to ER stress-induced apoptosis.

In summary, our data indicate that β-cells lacking IRS1 demonstrate low nuclear accumulation of sXBP-1, enhanced phosphorylation of eEF2, and elevated Ca^2+^ storage in ER which together contribute to resistance to ER-stress induced apoptosis (Fig. 6). Tissue specific downregulation of IRS1 by using chemical compounds such as NT157, known to inhibit insulin receptor substrates^52^, is one potential approach to protect β-cells from ER-stress induced cell death.

## Methods

### Preparation of β-cell lines

β-cell lines from control, IRS1KO or IRS2KO mice were generated as described previously^4^,^18^. Cells were maintained in DMEM media containing 25 mM glucose, supplemented with 10% FBS. Experiments were performed using 80–90% confluent cells.

### Western Blotting

Cells were solubilized in RIPA buffer as reported earlier and protein concentration was measured using a BCA protein assay kit (Pierce)^2^. Samples were resolved on SDS-PAGE. Proteins were transferred onto polyvinylidene difluoride membranes. The membranes were blocked in blocking buffer (Thermo Scientific), and incubated with primary antibodies overnight at 4 °C. XBP1 antibody was from Santa Cruz. IRE1α, Insulin receptor substrate 2 (IRS2), p-Eef2, Eef2, Lamin B1, β-actin and cleaved caspase 3 antibodies were from Cell Signaling. pIRE1α antibody was from Novus Biologicals. PERK antibody was from Rockland. α-tubulin and SERCA2 antibodies were from abcam. Insulin receptor substrate 1 (IRS1) antibody was from Millipore. Phospho-XBP1 (Ser61 and Thr48) antibodies were a kind gift from Umut. Ozcan MD (Children’s Hospital, Boston)^24^. Relative protein amounts were determined by ImageJ software (NIH).

### Co-immunoprecipitation

β-cells transfected with 3×Flag-tagged sXBP1 and V5-tagged ubiquitin were lysed with RIPA buffer. The lysate was centrifuged at 12,000 rpm for 30 min at 4 °C. Supernatant was collected and rotated overnight with anti-DYKDDDDK (Flag) Beads (Clontech) at 4 °C. Beads were washed three times with TBS. Proteins were eluted into SDS sample buffer by heating for 10min at 95 °C. Proteins were resolved on SDS-PAGE and detected by immunoblotting.

### Free fatty acid treatment

Palmitate were purchased from Sigma. Cells were incubated in medium with 0.5 mM palmitate plus 0.5% (w/v) BSA or 0.5% (w/v) BSA alone as a control. The preparation of the free fatty acid media has been described elsewhere^53^.

### RNA isolation and quantitative RT-PCR

Total RNA was extracted using RNeasy Mini Kit (QIAGEN). One μg RNA was reverse-transcribed using a high-capacity cDNA Archive Kit (Applied Biosystems). Quantitative PCR was performed in an ABI 7900HT system, using SYBR Green Master mix (Biorad). TBP was used as an internal control. Primers described previously^22^ were used for amplification.

### Apoptosis assay

Apoptosis was measured by Annexin V staining using an Annexin V-FITC Apoptosis kit (Sigma). Cells were stained according to the manufacture’s instruction and processed for flow cytometry (MACSQuant analyzer, Miltenyi Biotec).

### Polyribosomal profiling (PRP) experiments

PRP was performed as previously described with modification^54^,^55^. Briefly, after incubation with 100 μg/mL cycloheximide (CHX) for 10 min at 37 °C, cells were washed in ice cold PBS containing CHX (50 μg/ml) and lysed in 300 μl polysome buffer (200 mM KCl, 20 mM TrisHCl [pH 7.4], 10 mM MgCl2, 1% Triton-X, 50 U/ml RNasin [Promega], 100 μg/mL CHX). The cell lysates were homogenized using a 23-gauge needle and incubated on ice for 10 min followed by centrifugation at 13,000 × g for 10 min at 4 C. Supernatant was layered onto a 10–50% sucrose gradient solution containing 20 mM Tris-HCl (pH 7.4), 10 mM MgCl2, 200 mM KCl, and 50 μg/ml CHX. The sucrose gradients were subjected to centrifugation at 4 °C in a Beckman SW-41Ti rotor at 39,000 rpm for 2 hours. A piston gradient fractionator (BioComp Instruments, Fredericton, Canada) was used to fractionate the gradients, and absorbance of RNA at 254 nm was recorded using an on-line UV monitor.

### Puromycin incorporation assay

Puromycin was added to the culture medium at 1 ug/mL for 15 minutes and cells were washed twice with cold PBS and lysed as described previously^56^. Western blotting was performed as described above.

### Ca^2+^ reporter assay

Cells were transduced with adenovirus at an MOI of 500 to express the ER Ca^2+^ reporter D1ER^57^ or the mitochondrial matrix Ca^2+^ reporter D3MT. Recordings were performed 2d (D1ER) or 3d (D3MT) post-transduction. Dynamic changes in cytosolic [Ca^2+^] ([Ca^2+^]c) and organellar [Ca^2+^] were measured simultaneously as previously described^58^,^59^. Cells were plated on 25 mm circular glass coverslips 1 day prior to recording. Immediately before recording, tissue culture medium was removed and the cells incubated for 30 minutes at room temperature in HBSS supplemented with 1 μM Fura-2 acetoxymethylester (Invitrogen), 2% FBS and 0.02% (vol/vol) Pluronic F-127. Cells were then transferred to the temperature controlled stage on a Nikon TE2000 microscope and maintained at 37 C. Pre-warmed solutions were constantly perfused through the recording chamber at a rate of 4 ml/min using a peristaltic pump. Metafluor software (Molecular Devices) was used to control Sutter filter changers, image acquisition using a Cascade 650 camera and image processing. Images were acquired at a rate of 1 series of frames every 10s. Fluorescence measurements were made using the following wavelengths: Fura 2 excitation at 340 nm and 380 nm with emission at 510 nm, D1ER and D3MT were excited at 440 nm with emission at 485 nm and 535 nm. An oil-immersion Nikon 40 x superfluor lens was used to acquire images. Data was exported from Metafluor and imported into Origin 15 software (OriginLab) for further processing, plotting and statistical analysis.

### Autophagy Assay

Cellular autophagy was measured using Cyto-ID Autophagy Detection Kit (Enzo Life Sciences) according to manufacturer’s protocol. Fluorescent images were recorded in a confocal mode with a Zeiss LSM 710 microscope.

### Statistics

Results were shown as means ± SE and analyzed with an unpaired two-tailed Student’s t-test or ANOVA and Tukey test as post-hoc test. Differences were considered significant at p < 0.05.

## Additional Information

**How to cite this article**: Takatani, T. *et al*. IRS1 deficiency protects β-cells against ER stress-induced apoptosis by modulating sXBP-1 stability and protein translation. *Sci. Rep.* **6**, 28177; doi: 10.1038/srep28177 (2016).

## Supplementary Material

Supplementary Information
