# Corrigendum: IRS1 deficiency protects β-cells against ER stress-induced apoptosis by modulating sXBP-1 stability and protein translation

**DOI:** 10.1038/srep34969

**Published:** 2016-10-10

**Authors:** Tomozumi Takatani, Jun Shirakawa, Michael W. Roe, Colin A. Leech, Bernhard F. Maier, Raghavendra G. Mirmira, Rohit N. Kulkarni

Scientific Reports 6: Article number: 28177; 10.1038/srep28177 published online: 07 05 2016; updated: 10 10 2016.

In this Article the legend for Figure 5 is incomplete.

“Ca^2+^ in cytosol was measured using Fura-2 and Ca^2+^ in ER was measured using FRET-based probe D1ER cameleon in the basal state and after thapsigargin stimulation (100 nM for 1000 sec). Representative Ca^2+^ measurements in cytosol and ER (left panel). Quantitative ER Ca^2+^ levels of the average value prior to thapsigargin stimulation (Basal) and the minimum value after addition of thapsigargin (Tg(+)) (right panel). Data are means ± SEM, n = 43 for control, n = 50 for IRS1KO, and n = 40 for IRS2KO β-cells. *P < 0.05, **P < 0.01”.

Should read:

“(a) Ca^2+^ in cytosol was measured using Fura-2 and Ca^2+^ in ER was measured using FRET-based probe D1ER cameleon in the basal state and after thapsigargin stimulation (100 nM for 1000 sec). Representative Ca^2+^ measurements in cytosol and ER (left panel). Quantitative ER Ca^2+^ levels of the average value prior to thapsigargin stimulation (Basal) and the minimum value after addition of thapsigargin (Tg(+)) (right panel). Data are means ± SEM, n = 43 for control, n = 50 for IRS1KO, and n = 40 for IRS2KO β-cells. *P < 0.05, **P < 0.01. (b) Immunoblot of SERCA2 and β-actin in control β-cells, IRS1KO β-cells, and IRS2KO β-cells incubated with vehicle or thapsigargin (100 nM) for 8 h. Data are means ± SEM, n = 4. *P < 0.05”.

