# Supplementary material for: IRS1 deficiency protects β-cells against ER stress-induced apoptosis by modulating sXBP-1 stability and protein translation
Source: Sci Rep. 2016 Jul 5;6:28177. doi: 10.1038/srep28177 (PMC4932502; doi:10.1038/srep28177)
Supplement: Supplementary Information [file srep28177-s1.pdf]

Supplementary Information

IRS1 deficiency protects β-cells against ER stress-induced apoptosis by modulating sXBP-1 stability and protein translation

Tomozumi Takatani[1,5], Jun Shirakawa[1,5], Michael W. Roe[2], Colin Leech[2], Bernhard Maier[3], Raghavendra A. Mirmira[3,4] and Rohit N. Kulkarni[1]

[1]Islet Cell and Regenerative Biology, Joslin Diabetes Center, Department of Medicine, Brigham and Women's Hospital, Harvard Stem Cell Institute, Harvard Medical School, Boston, MA.

[2]Department of Medicine, State University of New York (SUNY), Upstate Medical University, Syracuse, NY

[3]Department of Pediatrics and Herman B Wells Center for Pediatric Research

[4]Department of Cellular and Integrative Physiology, Department of Biochemistry and Molecular Biology, Department of Medicine, Indiana University School of Medicine, Indianapolis, IN

[5]These authors contributed equally to this work

***Corresponding author**:
Rohit N. Kulkarni, MD, PhD,
Islet Cell and Regenerative Biology, Joslin Diabetes Center,
One Joslin Place, Boston, MA 02215, U.S.A.
Tel: +1-617-309-3460; Fax: +1-617-309-3476
E-mail: rohit.kulkarni@joslin.harvard.edu

Supplementary Fig. S1

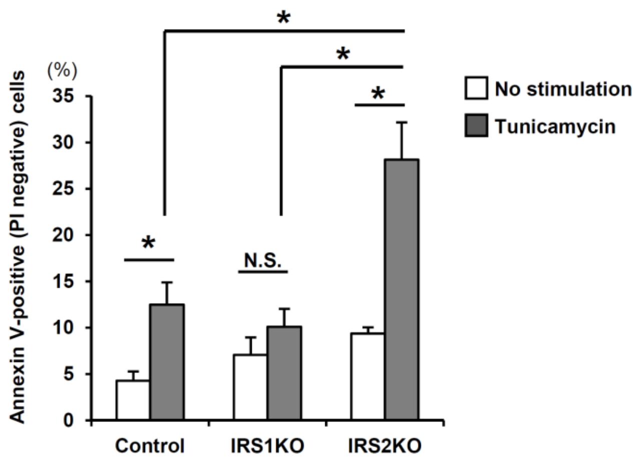

Supplementary Fig. S1. IRS1KO β-cells show resistance to tunicamycin-induced apoptosis

Results of FACS analysis for Annexin V-positive cells of control, IRS1KO, or IRS2KO β-cells incubated with tunicamycin (100 nM) for 24 h. Data are means ± SEM, n=3. **P* <0.05.

Supplementary Fig. S2

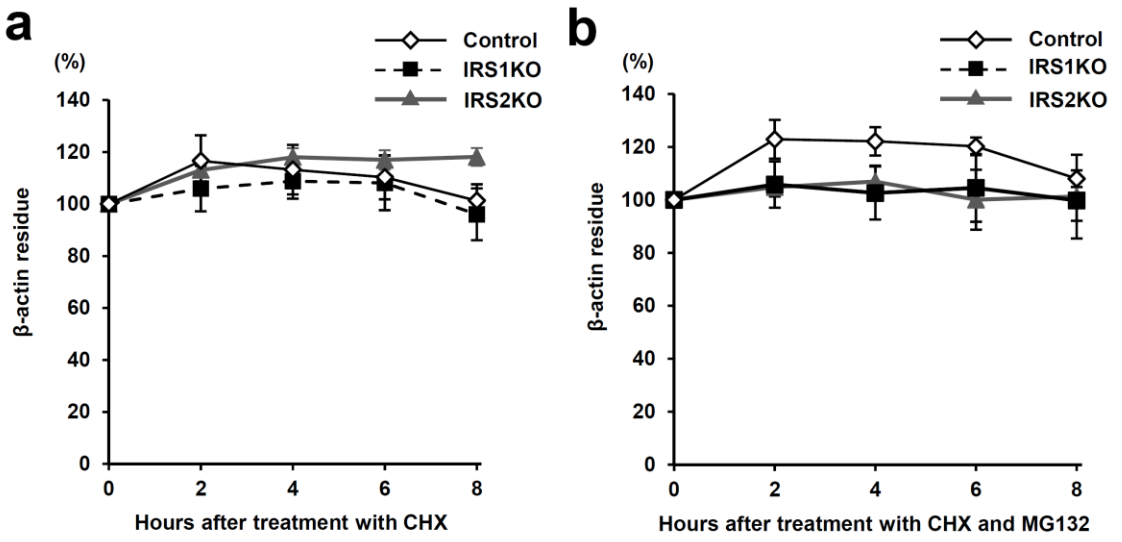

Supplementary Fig. S2. Stability of β-actin is similar in control, IRS1KO, or IRS2KO β-cells.

(a) CHX assay of β-actin using control, IRS1KO, or IRS2KO β-cells in Fig. 3c and 3d. Cells transfected and expressing sXBP-EGFP fusion protein were treated with CHX (100 μg/ml) and harvested at indicated times. Data are means ± SEM, n=3. (b) CHX assay of β-actin performed with MG132 (5μM) in Fig. 3f and 3g. Data are means ± SEM, n=3.

Supplementary Fig. S3

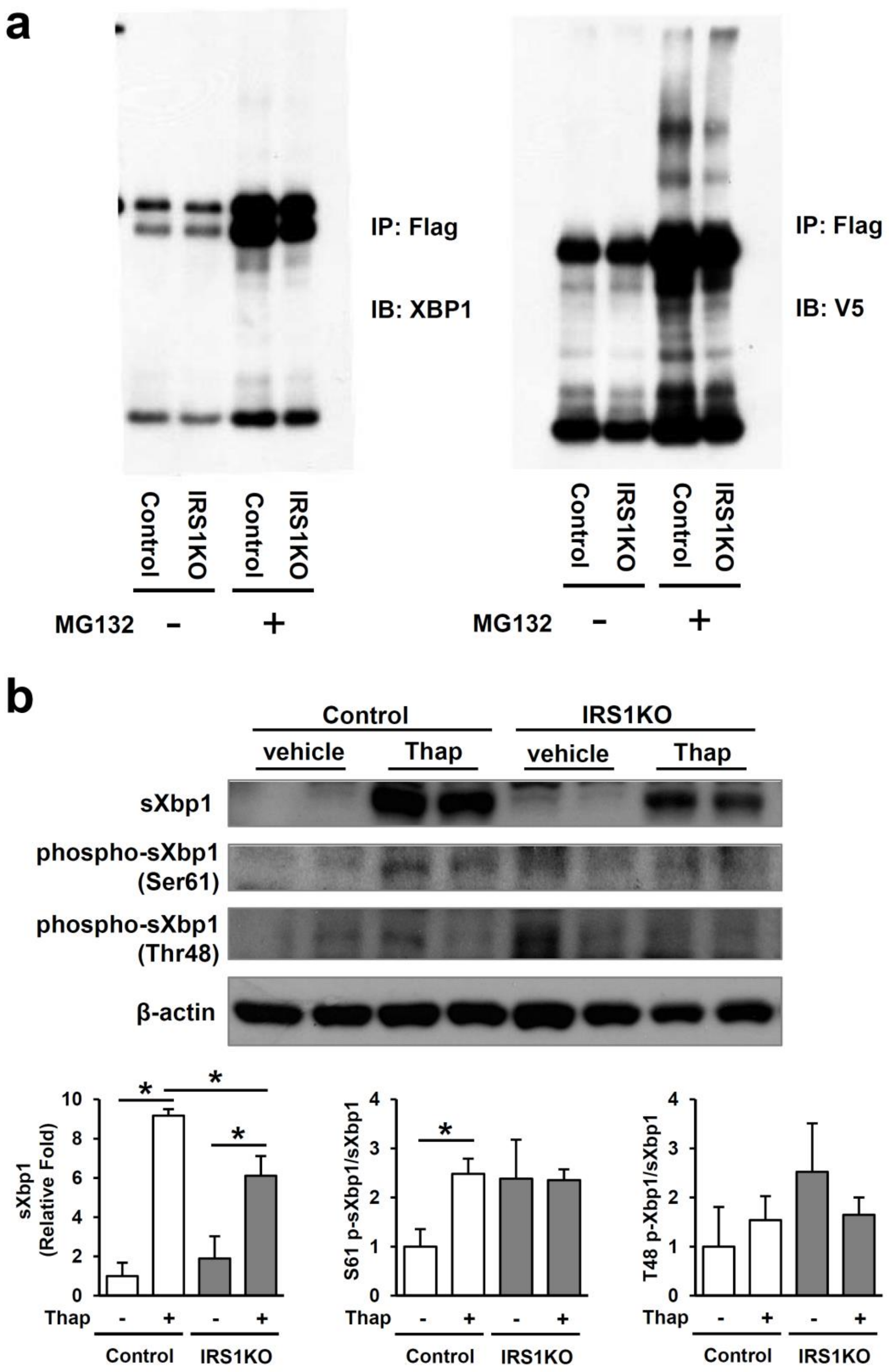

Supplementary Fig. S3. Ubiquitination and phosphorylation of sXBP1 in control and IRS1KO β-cells

(a) Control and IRS1KO β-cells were transfected with sXBP1-Flag fusion and ubiquitin-V5 fusion and treated with MG132 (5 μM). Cells were harvested and immunoprecipitated with anti-flag beads. Immunoblot was performed with anti-XBP1 or anti-V5 antibody. (b) Immunoblot of sXBP1, Ser61 phospho-sXBP1, and Thr48 phosphp-sXBP1 in control and IRS1KO β-cells incubated with vehicle (DMSO) or thapsigargin (100 nM) for 8h. Data are means ± SEM, n=4. *P <0.05.

Supplementary Fig. S4

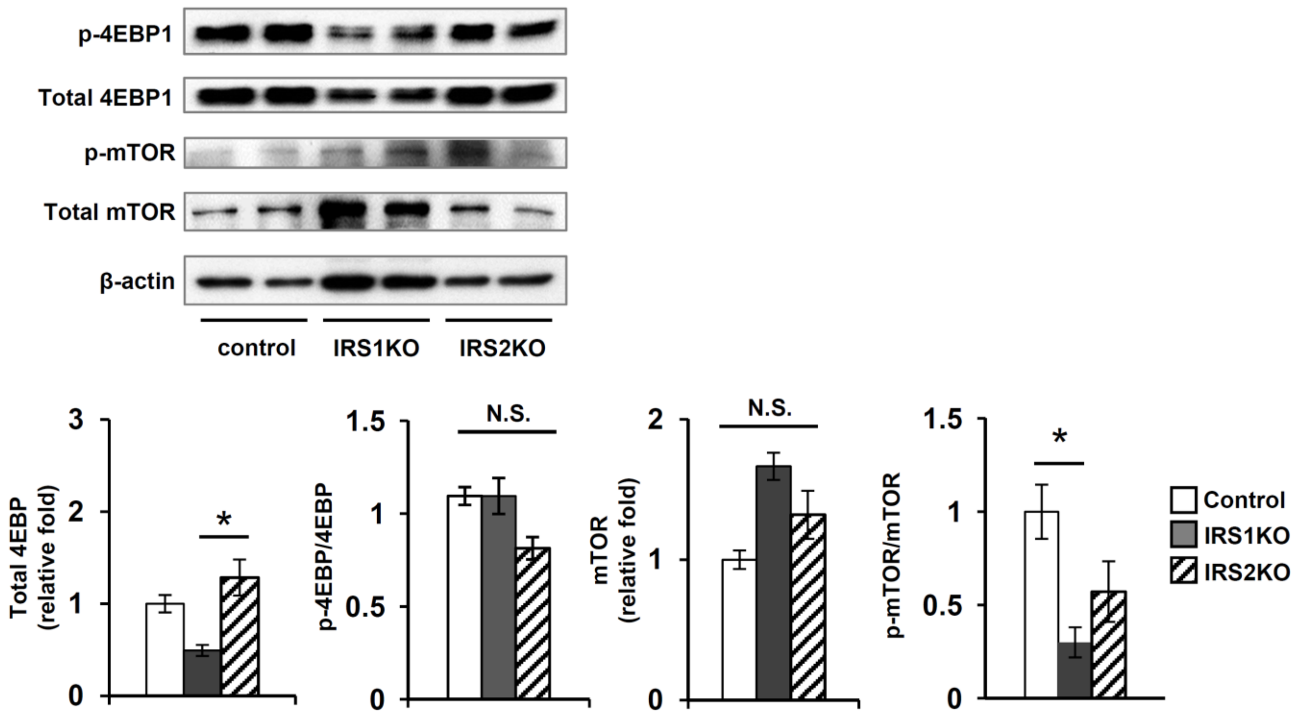

Supplementary Fig. S4. 4EBP-1 and mTOR expression and phosphorylation in control, IRS1KO and IRS2KO β-cells

Immunoblot of phospho-4EBP1, 4EBP1, phospho-mTOR, mTOR and β actin in control, IRS1KO and IRS2-KO β-cells in the basal state. Data are means ± SEM, n=4. **P* <0.05.

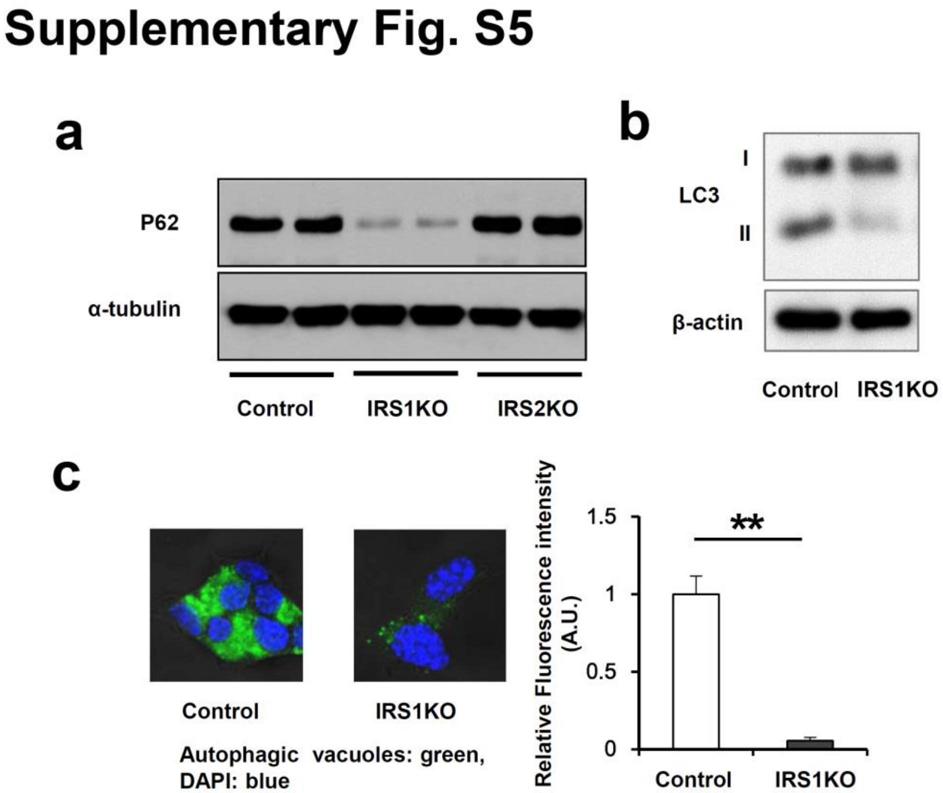

Supplementary Fig. S5. Altered expression of markers of autophagy in IRS1 KO β-cells

(a) Immunoblot of p62 and α tubulin in control, IRS1KO, or IRS2KO β-cells in the basal state. (b) Immunoblot of LC3 and β actin in control, IRS1KO, or IRS2KO β-cells in the basal state. (c) Representative immunofluorescence images of autophagy in control and IRS1KO β-cells. Cells were stained with Cyto-ID™ autophagic green fluorescent dye and viewed under a confocal microscope. Fluorescence signals in cells were measured using image J software. Data are means ± SEM, n=5. ***P* <0.01.

Supplementary Fig. S6

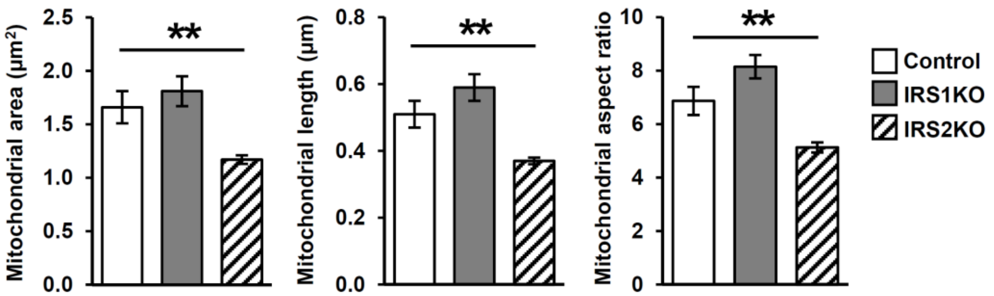

Supplementary Fig. S6. Mitochondorial morphology in control, IRS1KO, and IRS2KO β-cells

Measurement of mitochondrial morphology labeled with dsRedMT. Data are means ± SEM, n=350 for control, n=669 for IRS1KO β-cells, n= 662 for IRS2KO β-cells. ***P* <0.01.
